# Lipid Droplet-Binding Protein TIP47 Regulates Hepatitis C Virus RNA Replication through Interaction with the Viral NS5A Protein

**DOI:** 10.1371/journal.ppat.1003302

**Published:** 2013-04-11

**Authors:** Dorothee A. Vogt, Grégory Camus, Eva Herker, Brian R. Webster, Chia-Lin Tsou, Warner C. Greene, Tien-Sze Benedict Yen, Melanie Ott

**Affiliations:** 1 Gladstone Institute of Virology and Immunology, San Francisco, United States of America; 2 Pathology Service, Veterans Affairs Medical Center, and Department of Pathology, University of California San Francisco, San Francisco, United States of America; 3 Liver Center, University of California, San Francisco, United States of America; 4 Heinrich-Pette-Institute, Leibniz Institute for Experimental Virology, Hamburg, Germany; 5 Department of Medicine, University of California San Francisco, San Francisco, United States of America; University of California, San Diego, United States of America

## Abstract

The nonstructural protein NS5A has emerged as a new drug target in antiviral therapies for Hepatitis C Virus (HCV) infection. NS5A is critically involved in viral RNA replication that takes place at newly formed membranes within the endoplasmic reticulum (membranous web) and assists viral assembly in the close vicinity of lipid droplets (LDs). To identify host proteins that interact with NS5A, we performed a yeast two-hybrid screen with the N-terminus of NS5A (amino acids 1–31), a well-studied α-helical domain important for the membrane tethering of NS5A. Our studies identified the LD-associated host protein, Tail-Interacting Protein 47 (TIP47) as a novel NS5A interaction partner. Coimmunoprecipitation experiments in Huh7 hepatoma cells confirmed the interaction of TIP47 with full-length NS5A. shRNA-mediated knockdown of TIP47 caused a more than 10-fold decrease in the propagation of full-length infectious HCV in Huh7.5 hepatoma cells. A similar reduction was observed when TIP47 was knocked down in cells harboring an autonomously replicating HCV RNA (subgenomic replicon), indicating that TIP47 is required for efficient HCV RNA replication. A single point mutation (W9A) in NS5A that disrupts the interaction with TIP47 but preserves proper subcellular localization severely decreased HCV RNA replication. In biochemical membrane flotation assays, TIP47 cofractionated with HCV NS3, NS5A, NS5B proteins, and viral RNA, and together with nonstructural viral proteins was uniquely distributed to lower-density LD-rich membrane fractions in cells actively replicating HCV RNA. Collectively, our data support a model where TIP47—*via* its interaction with NS5A—serves as a novel cofactor for HCV infection possibly by integrating LD membranes into the membranous web.

## Author Summary

Hepatitis C Virus (HCV) belongs to the Flaviviridae family, and is an enveloped, positive, single-stranded RNA virus containing a 9.6 kb genome. Plus-strand RNA viruses induce a highly regulated process of membrane rearrangements and novel vesicle formation in infected cells (the “membranous web” for HCV) to create a suitable environment for RNA replication as well as for the assembly and release of new virions. HCV assembly occurs close to lipid droplets (LDs), cell organelles involved in fat storage and utilization. The viral non-structural protein NS5A plays a critical role in both viral RNA replication that occurs within the membranous web and viral assembly at LDs. We identified the host protein TIP47 as a novel host interaction partner for NS5A. TIP47 is a LD-binding protein also known to function as cargo in late-endosome-to-Golgi vesicular transport. Our data support a model where the recruitment of TIP47 by NS5A is required for viral RNA replication, indicating that LDs play a previously unrecognized role not only in viral assembly but also in RNA replication.

## Introduction

Plus-strand RNA viruses induce a highly regulated process of membrane rearrangements and novel vesicle formation in infected cells, in order to create a suitable environment for viral RNA replication as well as assembly and release of new virions. These viruses replicate their genome in membrane-associated complexes; however, the origin of the membranes used for replication varies from virus to virus. Infection with Hepatitis C Virus (HCV), a single- plus-stranded RNA virus within the Flaviviridae family, triggers rearrangements of intracellular membranes, resulting in membranous vesicles of heterogeneous size and morphology [1]. This so called “membranous web” is known to include ER membranes [2] and is considered to be the site of HCV RNA replication [3]. Expression of the non-structural viral protein NS4B in mammalian cells leads to membrane alterations resembling the membranous web [1]. However, molecular details of how the membranous web is formed in HCV-infected cells are still lacking.

The viral non-structural protein NS5A is a proline-rich, hydrophilic phosphoprotein that functions as a key regulator of HCV RNA replication and assembly [4]. NS5A has recently emerged as a major drug target in HCV infection, although the precise mode-of-action of NS5A-targeting drugs is not fully understood [5]. NS5A has no intrinsic enzymatic activity and is thought to function mainly as an adaptor protein for a wide variety of host proteins, including factors involved in host signaling, membrane trafficking, and lipoprotein synthesis pathways [6]–[8].

At the ER, NS5A is a component of the HCV replication complex that includes the two viral proteases NS2 and NS3/4A, the virally encoded RNA-dependent RNA polymerase NS5B, and the NS4B protein [1]. The N-terminus of NS5A forms a 30-amino-acid amphipathic α-helix that is highly conserved among HCV isolates [9]. Disruption of the amphipathic nature of the α-helix abolishes NS5A's membrane localization and viral replication [9], [10]. NS5A's α-helix is composed of a hydrophobic face embedded in the cytoplasmic leaflet of the ER membrane, and a polar-charged face exposed to the cytosol that is thought to mediate protein-protein interactions essential for the formation of a functional HCV replication complex [11].

However, no host protein that selectively interacts with the N-terminus of NS5A has yet been identified. In addition, NS5A binds to viral RNA as well as to numerous host proteins, and co-localizes with the viral capsid core in close proximity to lipid droplets (LDs), the site of HCV virion assembly [12]–[15]. NS5A has therefore been proposed to function as a transport vehicle that brings newly synthesized RNA from RNA replication sites to LDs for encapsidation, thus fulfilling critical functions in RNA replication and viral assembly [12], [13].

LDs are cytosolic lipid storage organelles consisting of neutral lipids (triacylglycerides and sterol esters) surrounded by a phospholipid monolayer and a growing list of associated proteins [16]. “**T**ail-**I**nteracting **P**rotein **47**” (TIP47) is a founding member of the LD-associated PAT (Perilipin, ADRP and TIP47) protein family that coats LDs and is involved in the regulation of LD generation and turnover [17]. TIP47 has been termed an “exchangeable” LD-associated protein, because it exists in other subcellular locations but moves to the surface of LDs upon rapid fat storage [18]. In contrast, ADRP when not bound to LDs is rapidly degraded *via* the ubiquitin/proteasome pathway [19], [20].

TIP47 was originally described to function as cargo in retrograde vesicular-membrane transport [21], [22], whereby mannose-6-phosphate receptors (MPRs) deliver newly synthesized lysosomal enzymes to endosomes and then recycle to the Golgi apparatus in cells. MPR recycling requires the Rab9-GTPase [23]. Rab9 recruits the cytosolic adapter TIP47 to late endosomes, a step that is thought to enhance its ability to bind MPR cytoplasmic domains during transport vesicle formation [21], [22]. These findings were recently disputed [24].

Because host proteins interacting with the N-terminus of NS5A have yet to be identified, we performed a yeast two-hybrid screen (Y2H) with this domain, which identified TIP47 as a novel interaction partner. TIP47 interacts with the NS5A N-terminus, and this interaction is required for efficient HCV RNA replication. Our data support a model where the TIP47/NS5A interaction serves to make LD membranes accessible for RNA replication, thus potentially participating in membrane rearrangements known to support HCV RNA replication.

## Results

### Interaction of the N-terminal amphipathic α-helix of NS5A with TIP47

**Figure 1:**
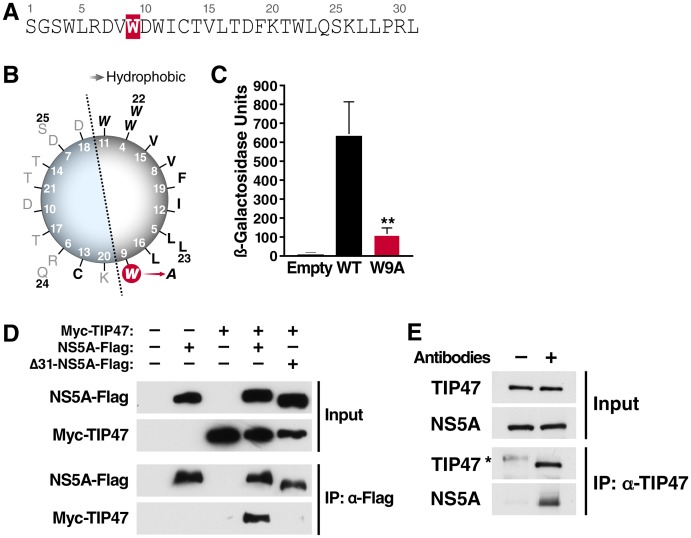
Yeast-two-hybrid screen identified TIP47 as a novel binding partner of NS5A. A) Sequence of the N-terminal 31 amino acids of NS5A (genotype 1b) that was used as bait in the screen. B) Wheel diagram of the N-terminal amphipathic α-helix of NS5A (amino acids 4–25). The position of the tryptophan-to-alanine mutation at position 9 (W9A) is highlighted in red. C) Quantitative Y2H assay of either wild-type NS5A (amino acids 1–14) as bait and control prey (empty), wild-type NS5A (amino acids 1–14) as bait and TIP47 as prey (WT), or NS5A containing W9A mutation (amino acids 1–4) as bait and TIP47 as prey (W9A). Values shown are means of β-galactosidase reporter gene activity ± standard deviation; n = 6, **P<0.01. D) Coimmunoprecipitation assays in Huh7 cells transfected with expression vectors for Myc-TIP47 and NS5A-Flag or Δ31-NS5A-Flag proteins. After immunoprecipitation with antibodies specific for Flag, TIP47 was detected by western blotting with antibodies specific for Myc. E) Coimmunoprecipitation assays in replicon Cells (Con1, genotype 1b, Figure 2A). After immunoprecipitation with antibodies specific for Flag (antibodies -) or TIP47 (antibodies +), TIP47 and NS5A were detected by western blotting with antibodies specific for TIP47 or NS5A, respectively. * unspecific band.

To identify host factors that interact with HCV NS5A, a Y2H assay was performed using the N-terminal amphipathic α-helix as bait (Figure 1A,B). The screen resulted in 22 confirmed positive clones. 12 of the 22 clones were found to be TIP47 (4 by sequencing and 8 by restriction digest), thus identifying TIP47 as a novel interaction partner with this domain in NS5A. Using a quantitative Y2H assay, the binding site of NS5A to TIP47 was mapped to a minimal domain spanning amino acids 1–14 in NS5A (Figure 1C). A tryptophan-to-alanine mutation at position nine (W9A) decreased protein binding by 6-fold compared to the wild-type sequence (Figure 1C), but did not interfere with the subcellular localization of NS5A when introduced into the full-length NS5A protein (Figure S1). These results are consistent with previous data indicating that the N-terminus of NS5A is involved in protein-protein interactions essential for HCV propagation independently from its reported role in membrane localization [11].

The TIP47-NS5A interaction was confirmed in coimmunoprecipitation experiments in human Huh7 hepatoma cells. Myc-tagged TIP47 was overexpressed together with FLAG-tagged NS5A, or a deletion mutant in which the N-terminal 31 amino acids were missing (Δ31-NS5A-Flag). TIP47 coimmunoprecipitated with full-length but not N-terminally truncated NS5A, confirming that these proteins interact in a manner dependent on the amphipathic α-helix of NS5A (Figure 1D).

**Figure 2:**
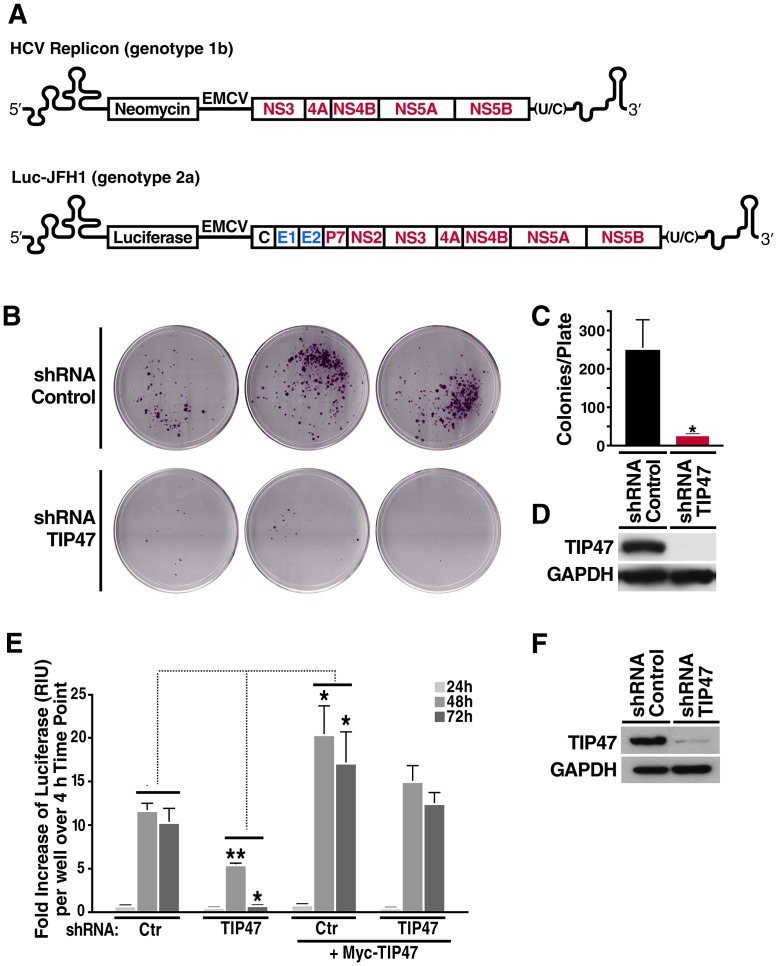
TIP47 is required for HCV RNA replication. A) Schematic representation of HCV Replicon (Con1, genotype 1b), a subgenomic bicistronic HCV replicon with neomycin as a reporter gene, and Luc-JFH1 (genotype 2a), a full-length bicistronic HCV infectious clone with firefly luciferase as a reporter gene. B) Crystal violet-stained Huh7.5 cells transfected with control shRNA or shRNA against TIP47 and HCV replicon RNA (A). G418 was added to the medium (1 mg/ml). Cells were kept under selective pressure for 4 weeks. Only cells actively replicating HCV RNA, and thus expressing the neomycin phosphotransferase survive. C) Quantification of colony numbers/plate in (B) (mean ± standard deviation; n = 3, *P<0.05). D) Western blot analysis of TIP47 expression in shRNA-expressing Huh7.5 cells at the time of transfection in (B). E) Transfection of shRNA-expressing Huh7.5 cells with Luc-JFH1 RNA (A) and Myc-TIP47 expression plasmid (where indicated). HCV replication was measured by determining the fold increase of luciferase (RLU) per well over a 4 h time point (mean ± standard deviation; n = 3; *P<0.05, **P<0.01). F) Western blot analysis of TIP47 expression in shRNA-expressing Huh7.5 cells at time of transfection in (E).

Furthermore, the TIP47-NS5A interaction was confirmed in the context of active HCV RNA replication in Huh7.5 cells harboring a subgenomic HCV replicon expressing only the non-structural viral proteins NS3–5B (Figure 1E). This replicon is sufficient to autonomously replicate HCV RNA but cannot support infectious particle production since critical structural proteins as well as p7 and NS2 are lacking (Figure 2A, HCV Replicon, genotype 1b) [25]). Collectively, these results identify TIP47 as a novel host factor binding to the N-terminus of NS5A.

### TIP47 is required for HCV RNA replication

To study TIP47's role in HCV infection, we introduced shRNAs directed against TIP47 or non-targeting control shRNAs into Huh7.5 cells *via* lentiviral transduction. No toxicity or significant alterations in LD content were observed in cells after TIP47 knockdown (Figure S2). Transduced cells were then transfected with replicon RNA (genotype 1b) and selected with G418. The replicon RNA encodes the neomycin phosphotransferase selection gene thus only cells actively replicating HCV RNA survive (Figure 2A). After four weeks, surviving cell clones were fixed, stained with crystal violet and counted. We found that down regulation of TIP47 expression resulted in a more than 10-fold decrease in colony formation as compared to control cells, indicating that TIP47 plays an important role in HCV RNA replication (Figure 2B, C). The loss of TIP47 expression was verified by western blotting (Figure 2D). These studies reveal that TIP47 functions as a new cellular regulator of HCV RNA replication.

We confirmed these results in the context of fully infectious HCV. Here, shRNA-transduced cells were transfected with infectious RNA of HCV-JFH1 (genotype 2a), carrying firefly luciferase as a reporter gene (Figure 2A, Luc-JFH1). Cells were harvested at different time points after transfection and subjected to luciferase assays. All values were expressed as the fold increase of luciferase (RLU) per well over an early 4-hour time point before RNA replication occurs [26], [27]. We observed a 10-fold decrease in luciferase ratios at 72 hours post-transfection when TIP47 expression was down regulated compared to control cells (Figure 2E,F). When we reconstituted TIP47 expression in shRNA-containing cells *via* overexpression of an shRNA-resistant allele, HCV infection was restored underscoring the specific effect TIP47 plays in HCV infection (Figure 2E). In accordance with these observations, over-expression of Myc-TIP47 in cells treated with control shRNAs enhanced HCV replication, pointing to a limiting function of TIP47 expression for HCV infection in cells (Figure 2E).

### TIP47 regulates HCV RNA replication *via* interaction with NS5A

**Figure 3:**
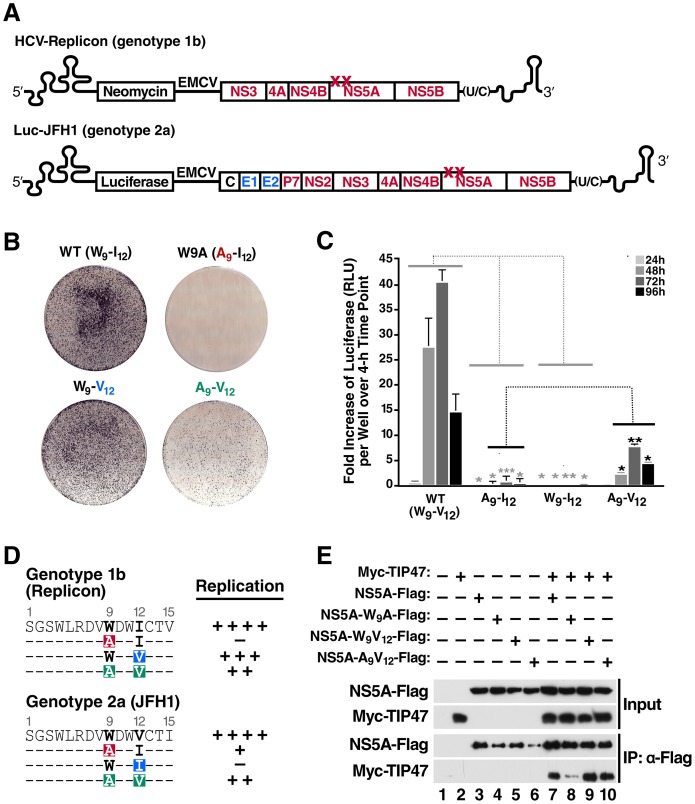
TIP47 is involved in HCV RNA replication *via* interaction with NS5A. A) Schematic representation of the HCV Replicon (genotype 1b) and Luc-JFH1 (genotype 2a), with mutations at position 9 and 12 in the NS5A N-terminus. B) Crystal violet-stained Huh7.5 cells transfected with wild-type or mutant HCV replicon RNA (genotype 1b). 24 h post-transfection, G418 was added to the medium (1 mg/ml). Cells were kept under selective pressure for 4 weeks before being stained with crystal violet. These results are representative of three independent experiments. C) Luciferase assay of Huh7.5 cells transfected with wild-type Luc-JFH1 RNA (genotype 2a) or Luc-JFH1 RNA containing indicated NS5A mutations. Values are expressed as fold increase of luciferase (RLU) per well, over a 4 h time point (mean ± standard deviation; n = 3, *P<0.05, **P<0.01, ***P<0.001). The decrease of A_9_-I_12_ and W_9_-I_12_ is significant in relation to WT (indicated in grey *), whereas the increase in A_9_-V_12_ is significant in relation to A_9_-I_12_ where indicated (indicated in black *). D) Amino acid sequences of the NS5A N-terminus of HCV replicon (genotype 1b) and HCV-JFH1 (genotype 2a). Positions 9 and 12 are highlighted in bold black and mutations are highlighted in color. Summary of respective replication phenotypes in the HCV replication assay (genotype 2a, Figure 3B, C). Wild-type replication phenotypes were set as equal to (++++) in their respective assays, while reduced replication levels were defined in the Replicon assay as (+++) for ∼90% of wild-type colonies, (++) for ∼20% colonies, or (−) for no colonies. In the Luciferase assay, luciferase values were described as (++) for values ∼19% of wild-type, (+) for ∼2%, and (−) for values below 1%. E) Coimmunoprecipitation assays in Huh7 cells transfected with expression vectors for Myc-TIP47 and NS5A-Flag or NS5A-Flag protein carrying either A_9_-I_12_ (W9A), W_9_-I_12_, or A_9_-V_12_ mutations. After immunoprecipitation with Flag antibodies, TIP47 was detected by western blotting with antibodies specific for Myc.

To examine whether the interaction with NS5A is important for TIP47's role in HCV RNA replication, we introduced the W9A mutation that decreased the NS5A-TIP47 interaction into the replicon cells, and transfected the mutated version into Huh7.5 cells as described above (Figure 3A, HCV-Replicon). The W9A mutation almost completely abolished colony formation in transfected Huh7.5 cells (Figure 3B, compare WT to W9A). To test for revertant or compensatory mutations, we sequenced the NS5A region in the few surviving colonies, and found that all sequenced clones maintained the W9A mutation, but discovered that all clones carried a second isoleucine-to-valine mutation at position 12 (I12V). To test whether the I12V mutation suppresses the RNA replication defect induced by the W9A mutation, the I12V mutation alone as well as the combined W9A-I12V mutation was cloned back into the wild-type replicon RNA and studied in HCV replicon assays. The I12V mutation alone resulted in similar colony numbers as the wild-type replicon RNA (Figure 3B, compare W_9_-V_12_ to WT). However, when combined with the W9A mutation, we found that the I12V mutation partially restored colony formation, suggesting that this mutation evolved to overcome the replication defect induced by the loss of TIP47 binding (Figure 3B, compare A_9_-V_12_ to W9A). Using full-length Luc-JFH1 constructs (Figure 3A, Luc-JFH1), we obtained similar results as with the HCV replicon: a near complete defect in RNA replication with the A_9_-I_12_ mutation and a partial rescue of this defect with the A_9_-V_12_ mutation (Figure 3C,D). However, it must be noted that the HCV genotype 2a NS5A protein contains valine rather than isoleucine at position 12 in its wild-type sequence (Figure 3D). Surprisingly, a valine to isoleucine mutation at position 12 alone was lethal in genotype 2a (Figure 3C,D, compare W_9_-I_12_ to WT). Analysis of the NS5A sequence from all genotypes shows that W9 is highly conserved while position 12 is occupied by an isoleucine only in genotype 1 [28].

Next, we tested the effect of these mutations on TIP47 binding to NS5A. Single and double mutations were cloned into the FLAG-tagged NS5A expression vector, transfected along with Myc-tagged TIP47 in Huh7 hepatoma cells, and subjected to immunoprecipitation and western blot assays to determine the extent of the interaction. While cells singly expressing either wild-type or I12V-mutant NS5A co-immunoprecipitated efficiently with TIP47, a significant decrease in binding was observed with the W9A mutant (Figure 3E, compare lane 7 with lane 8), consistent with the results obtained in the Y2H assays (Figure 1C). In contrast, TIP47 binding was restored in cells expressing the double-mutant NS5A construct, indicating that the I12V mutation rescued HCV RNA replication by re-establishing the interaction with TIP47 (Figure 3E, lane 10). These results underline the importance of the NS5A-TIP47 interaction for HCV RNA replication.

### Altered membrane association of TIP47 during HCV RNA replication

**Figure 4:**
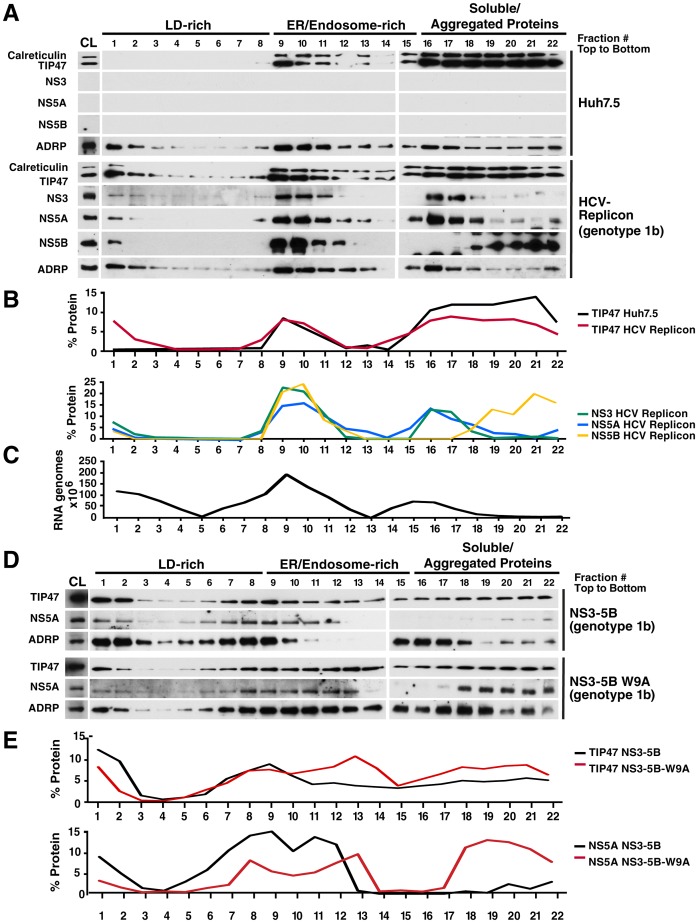
TIP47 cofractionates with lipid droplets, viral nonstructural proteins, and viral RNA during HCV RNA replication. A) Western blot following the membrane flotation assay in an iodixanol gradient. Briefly, cytoplasmic lysates (CL) of either naïve Huh7.5 cells or Huh7.5 cells harboring a subgenomic HCV replicon (genotype 1b) were mixed 1∶1 with 60% iodixanol (in PBS/0.25 M sucrose) for a final 30% iodixanol concentration, then overlaid with 20% and 10% iodixanol. The gradient was spun at 200,000 g for 16 h at 4°C. 22 fractions were collected from top to bottom, and then assessed by western blot with antibodies specific for calreticulin, TIP47, NS3, NS5A, NS5B, or ADRP. Western Blots are representative of 4 independent experiments. B) Profile of protein expression from western blot in (A). Protein expression is shown as protein amount (as measured by intensity) in one fraction as a percentage of total protein amount in all 22 fractions combined. Protein quantification was done in ImageJ. C) Amount of viral RNA genomes in each fraction in replicon cells in (A) as determined by quantitative RT-PCR. D) Western blot following the membrane flotation assay in an iodixanol gradient of Huh7.5 cells transfected with an expression vector of either wild-type NS3-5B (genotype1b) or containing a W9A mutation in NS5A. Crude Lysate (CL) was prepared 24 h post-transfection, and subjected to gradient centrifugation. 22 fractions were collected from top to bottom, and then assessed by western blot with antibodies specific for calreticulin, TIP47, NS5A, or ADRP. E) Profile of protein expression from western blot in (D). Protein expression is shown as protein amount (as measured by intensity) in one fraction as a percentage of total protein amount in all 22 fractions combined. Protein quantification was done in ImageJ.

As HCV RNA replication occurs within the membranous web, we next examined whether TIP47 associates with HCV replicase-containing intracellular membranes. We performed membrane flotation assays in naïve or HCV replicon-containing Huh7.5 cells (genotype 1b), in which membrane compartments of crude cell lysates (CL) were separated in a self-forming linear 10–20–30% iodixanol density gradient as described [29]. Twenty-two fractions were collected from top to bottom and assessed by western blotting for TIP47 expression. Cell lysates were also analyzed by western blotting before gradient centrifugation to assess the overall cellular expression of the proteins. No significant differences in expression levels were found for TIP47 in the lysates of HCV replicon–expressing cells compared to Huh7.5 control cells Figure 4A, CL).

We divided the 22 fractions obtained from the gradient into three areas based on their membrane-associated protein content. The lowest-density membrane fractions (fractions 1–8) were rich in ADRP, a constitutive LD-binding protein [30], but devoid of calreticulin, an integral ER marker, and were therefore labeled “LD-rich” (Figure 4A). Fractions 9–15 contained calreticulin and endosomal Rab proteins (Figure S2A, B) as well as ADRP and were labeled “ER/endosome-rich”, while fractions 16–22 contained soluble and aggregated proteins as previously determined [31] (Figure 4A).

In naive Huh7.5 cells, TIP47 was mainly found in the ER/endosome-rich and the soluble/aggregated protein fractions, but not within low-density LD-rich membranes (Figure 4A, upper 5 panels). This distribution changed in HCV replicon–containing cells, where TIP47 was found in low-density LD-rich membranes (Figure 4A, lower 5 panels). Overall the fractions of membrane-bound TIP47 increased—especially in LD-rich fractions, with about 7% of total TIP47 in fraction 1 alone—while soluble TIP47 levels decreased (Figure 4B). A slight increase of TIP47 in ER/endosome-rich fractions 9–11 was also observed (Figure 4B). In contrast, the subcellular distributions of LD- and ER-specific markers, ADRP and calreticulin, did not change significantly upon HCV RNA replication, except for a slight increase in calreticulin in fraction 1 (Figure 4A). These results indicate that in cells that actively replicate HCV, TIP47's membrane association is enhanced with enrichment in LD-rich membranes.

### Cofractionation of TIP47 with viral RNA and NS5A, NS5B, and NS3 proteins

To test whether TIP47 cofractionated with HCV replication complexes, we blotted the gradient fractions with antibodies against HCV NS5A, NS5B, and NS3 proteins. In HCV replicon–containing cells, the viral proteins NS3, NS5B, and NS5A cofractionated with TIP47 in ER/endosome-rich fractions, and like TIP47, were also found in LD-rich fractions, most prominently in fraction 1 (Figure 4A,B). As all three viral proteins are part of the HCV replication complex, this finding supports a model in which TIP47 associates with HCV RNA replication complexes both within the ER and at LD-rich membranes. In support of this model, viral RNA was detected in LD- and ER/endosome-rich fractions with highest concentrations in fractions 1 and 9 underscoring that these fractions harbor actively replicating replicase complexes (Figure 4C).

We also performed similar membrane flotation assays in Huh7.5 cells expressing the NS3–5B polyprotein from a DNA-based expression vector, which allowed us to compare wild-type and W9A mutant NS5A in the context of the HCV replicase independently from RNA replication. Redistribution to LD-rich membranes was reduced for both, TIP47 and NS5A, when the NS5A W9A mutant was tested, indicating that the joint association with these membranes depends, at least in part, on efficient NS5A-TIP47 interactions (Figures 4D, E).

As TIP47 has been implicated as an adaptor protein in late-endosome-to-Golgi trafficking, we also tested the distribution of several endosomal Rab GTPases in the gradient fractions, including the previously described TIP47 interaction partner Rab9 [21], [22]. Rab9 was not found in LD-rich fractions in control cells, and showed only a modest (3% of all protein) enhancement in fraction 1 in HCV replicon–expressing cells (Figure S3A, B). In contrast, the early endosomal protein Rab5 was found in LD-rich fractions in uninfected and HCV replicon-containing cells, consistent with previous reports demonstrating its association with the surface of LDs [32] (Figure S3A, B). In HCV replicon-containing cells, a strong redistribution to LD-rich fractions was observed for Rab7, a late endosomal GTPase previously shown to play a critical role in HCV RNA replication [26], [33] (Figure S3A, B). Interestingly, we noted a marked (∼3-fold) overall increase in Rab9 expression in the cell lysates of HCV replicon–containing cells compared to Huh7.5 control cells, suggesting enhanced late-endosome-to-Golgi trafficking in HCV replicon–expressing cells (Figure S3A).

### Association of TIP47 with LDs during HCV infection

**Figure 5:**
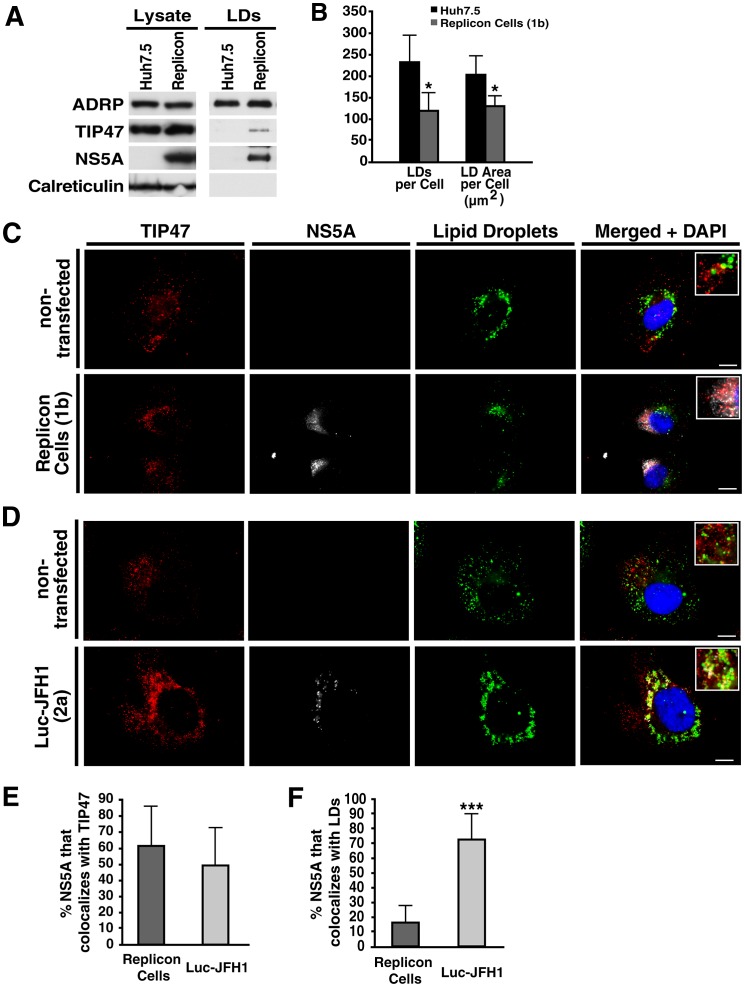
TIP47 associates with LDs during HCV infection. A) Western blot analysis of cell extracts or isolated lipid droplet fractions from either naïve Huh7.5 or replicon Cells (genotype 1b). B) Quantification of number of LDs (RedO stain) and LD area per cell in either naïve Huh7.5 or replicon cells (genotype 1b) (mean ± standard deviation; n>100, *P<0.05). Quantification was done using Volocity software (Perkin Elmer). C) Indirect immunofluorescence of TIP47 (red) and NS5A (white) in either non-transfected Huh7.5 cells, or replicon Cells (genotype 1b), Lipid droplets were stained with Bodipy493/503 (green). The scale bar = 10 µm. The close-up images are shown with 2.5 fold higher magnification. D) Indirect immunofluorescence of TIP47 (red) and NS5A (white) in either non-transfected Huh7 Lunet cells, or transfected with full-length Luc-JFH1 (genotype 2a). Lipid droplets were stained with Bodipy493/503 (green). The scale bar = 10 µm. The close-up images are shown with 2.5 fold higher magnification. E) Quantification of the percentage of NS5A that colocalizes with TIP47 in either Replicon-expressing cells or Huh7 Lunet cells transfected with Luc-JFH1 (as seen in B) (mean ± standard deviation; n≥30). Quantification was done with the automatic colocalization program in Volocity software (Perkin Elmer). F) Quantification of the percentage of NS5A that colocalizes with LDs in either replicon-expressing cells (as seen in C) or Huh7 Lunet cells transfected with Luc-JFH1 (as seen in D) (mean ± standard deviation; n≥30, ***P<0.001). Quantification was done with the automatic colocalization program in Volocity (Perkin Elmer).

To independently study the association of TIP47 with LDs during HCV replication, we isolated highly purified LD fractions free of ER contaminations from naïve Huh7.5 or replicon cells. These studies confirmed that TIP47, together with NS5A, was selectively recruited to purified LD fractions in replicon cells but was not present at LDs in naïve Huh7.5 cells (Figure 5A). As TIP47 is known to associate with LDs during active LD formation, we examined the LD content in replicon cells. Of note, replicon–containing cells do not express the viral protein core, known to induce LD accumulation in infected cells [34], [35]. Contrary, we found that the number of LDs per cell was significantly down regulated in replicon cells in comparison to naïve Huh7.5 cells excluding the possibility that enhanced LD formation triggered the translocation of TIP47 to LDs in replicon cells (Figure 5B).

In indirect immunofluorescence microscopy, more than 60% of NS5A colocalized with TIP47 in replicon cells (genotype 1b) (Figures 5C, E). Interestingly, only ∼15% of NS5A colocalized with LDs in these cells, confirming results from membrane flotation assays showing that the majority of NS5A and TIP47 are found at ER/endosome-rich membranes or are soluble (Figures 5C, F). Interestingly, the recruitment to LDs was enhanced in cells expressing full-length JFH1 virus (genotype 2a) with ∼50% of NS5A colocalizing with TIP47 and the majority of the protein found at LDs (Figures 5D–F). This is likely due to the steatogenic properties of the viral core protein expressed in the full-length virus but not in the replicon-expressing cells [34], [35]. In non-transfected cells, no significant association of TIP47 with LDs was observed, confirming previous reports and our own findings in membrane flotations and LD isolation assays as described above (Figures 4A, B, 5A) [18].

In contrast, no colocalization of Rab9 with NS5A or LDs was observed in cells infected in a single-round-infection with the Jc1 strain of HCV (Figure S3C). In naive and transfected cells, Rab9 showed the same highly concentrated localization to one perinuclear area, likely late endosomes, that did not overlap with LDs. NS5A colocalized with LDs in perinuclear areas surrounding punctate Rab9-containing subcellular regions, but no actual colocalization between Rab9 and NS5A was observed (Figure S3C). Thus, Rab9 appears not involved in the newly described HCV cofactor role of TIP47 and supports the model that the LD-binding rather than the vesicular transport properties of TIP47 are required to support HCV RNA replication.

## Discussion

In the present study, we identified TIP47 as a novel host factor that interacts with the N-terminal amphipathic α-helix of NS5A. Previous studies suggested that this protein domain, in addition to being the membrane anchor for NS5A, is also involved in protein-protein interactions that are essential for HCV RNA replication [11]. In a quantitative Y2H assay, we showed that a single point mutation (W9A) in NS5A –previously implicated in the protein:protein interactor function of the domain– significantly decreased binding between the amphipathic α-helix and TIP47, supporting the model that this domain is a critical interaction domain with host factors in NS5A and that cellular TIP47 is a *bone fide* interaction partner. The W9A point mutation is located at the hydrophobic face of the helix, which is predicted to contact the cytoplasmic leaflet of the ER membrane, indicating that the membrane-binding face of the helix, rather than the polar-charged cytosolic side, mediates TIP47 interaction. The interaction was confirmed in coimmunoprecipitation assays in the context of actively replicating HCV.

Two independent sets of experiments support the model that TIP47 plays a critical role in HCV RNA replication. First, shRNA-mediated knockdown of TIP47 markedly decreased viral propagation of full-length infectious clones of HCV as well as viral RNA replication in hepatoma cells containing subgenomic replicons. The fact that a similar degree of suppression was observed in both viral systems is consistent with the notion that RNA replication is the major step in the viral life cycle affected by TIP47. However, we cannot exclude that other steps in the viral life cycle may also depend on functionally intact TIP47. These include particle assembly known to require LDs [12].

The second line of evidence supporting a critical role for the TIP47-NS5A interaction in HCV infection comes from replication studies of mutant HCV replicons. The W9A mutation we used was previously described to leave the amphipathic structure of the N-terminal α-helix in NS5A intact, but was shown to cause significant HCV RNA replication defects [11]. We confirmed and extended these studies by identifying TIP47 as a cellular factor that binds to the NS5A amphipathic α-helix in a W9-dependent manner. We also identified I12V as a compensatory mutation that restored NS5A binding and partially restored the replication deficit when introduced into W9A-mutant NS5A. Since amino acids 9 and 12 are predicted to be “neighbors” in the α-helical structure of the NS5A N-terminus (with 3.6 residues per turn), we hypothesize that both residues are important for TIP47 binding.

In both genotypes (1b and 2a), a combination of A_9_-I_12_ in NS5A was detrimental to replication, while the A_9_-V_12_ combination partially rescued replication. Why the suppressor mutation at position 12 restores binding to W9A-mutant NS5A to near wild-type levels, but only partially restores the RNA replication defect, remains unclear. A possible explanation is that W9 is involved in NS5A functions other than TIP47 recruitment and that these functions cannot be compensated by the I12V mutation.

**Figure 6:**
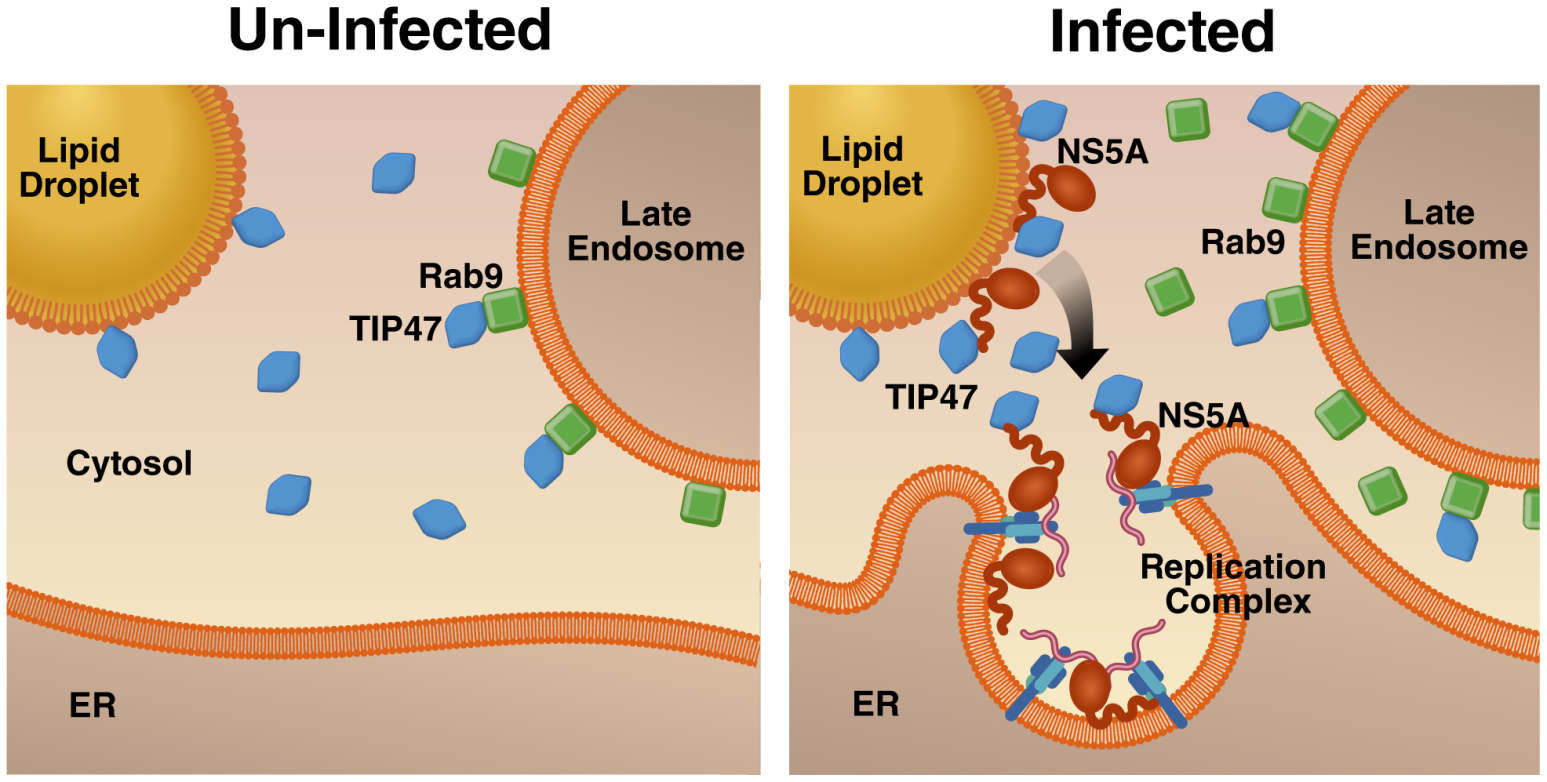
Model: TIP47 recruits LD membranes to the membranous web *via* interaction with NS5A. **In uninfected cells**: TIP47 is involved in the retrograde trafficking pathway by interacting with Rab9 at late endosomes, and functions as an “exchangeable” LD-associated protein that is mostly present in the cytosol but moves to LDs upon rapid fat storage. **In HCV-infected cells**: TIP47 interacts with NS5A-containing replication complexes at the ER and NS5A at LDs, thus making LD membranes accessible for HCV RNA replication for either integration of LDs into the membranous web, or as source of energy for the creation of the membranous web. HCV infection also increases endosome-to-Golgi trafficking *via* the up-regulation of Rab9 expression.

A striking finding in our study is that LD membranes are directly linked to viral RNA replication. It has been proposed that the HCV core protein brings ER membranes in close proximity to LDs to create a localized environment with a high concentration of membranes for viral replication and assembly [12], [36]. Here, we show that a close LD/ER membrane interface is also established in the absence of viral structural proteins, most notably in the absence of core. Core is known to coat the surface of LDs, a critical step for the recruitment of viral RNA-replication complexes to the vicinity of LDs, and this process is essential for the encapsidation of viral RNA into progeny virions [12]–[15]. The finding that viral NS3, NS5B, and NS5A proteins together with TIP47 and viral RNA access LD membranes in the absence of core points to LD membranes as critical parts of the membranous web independent from viral assembly (Figure 6). This model is supported by the finding that Rab7, a GTPase previously shown to associate with HCV replication complexes, is also newly distributed to LD membranes in HCV replicon-containing cells, underscoring the relevance of these membranes for HCV RNA replication.

How this redistribution is orchestrated and whether the LD-binding functions of TIP47 or NS5A are important for this process, remain unknown. Our experiments in cells expressing the HCV replicase containing wild-type or W9A mutant NS5A underscores that the TIP47/NS5A interaction is participating in this process. Our observation that LD content is reduced in HCV replicon cells as compared to parental cells is interesting and suggests that LDs are being consumed during HCV replication, as a potential source for membranes or energy. This may explain why the colocalization between NS5A and LDs is relatively low in replicon cells as compared to cells infected with full-length HCV as the overall LD content is reduced. As LDs are overall upregulated in cells infected with full-length HCV [34], [35], this consumption is likely counterbalanced in the presence of structural genes, most notably core, to provide select assembly “platforms” to enable productive HCV virion formation. A similar “consumption” of LDs during viral infection was recently described for Dengue virus infection and involved autophagy [37].

This is the first demonstration of TIP47's involvement in viral RNA replication and LD consumption. Previous studies have linked the host factor to assembly roles in other viral life cycles, such as HIV, Dengue, and Vaccinia virus. In HIV, TIP47 functions as a connector protein by binding the cytoplasmic domain of ENV and the matrix (MA) domain of Gag [38]-[40]. Similar to the NS5A membrane anchor in our study, the TIP47-binding region in MA overlaps a hydrophobic region implicated in Gag anchoring and binding at the plasma membrane [38]–[40]. It is therefore possible that TIP47 binding to viral factors serves to compete with membrane anchoring of host binding partners, thus aiding in their release from membrane attachment and facilitating relocalization to other membranes and/or interaction with other proteins.

In Dengue virus infection, TIP47 was shown to be required for the Dengue capsid protein to associate with LDs and promote viral assembly [41], [42]. In contrast, in Vaccinia virus, the viral p37 protein was shown to associate with TIP47-, Rab9-, and MPR-containing membranes, and mutations in p37 that block association with TIP47 were shown to inhibit plaque formation [43]. These studies indicated that p37 interacts with proteins associated with late endosome–derived transport vesicles to facilitate Vaccinia viral assembly [43].

During HCV infection, Rab9 is only moderately redistributed to LD-rich, TIP47- and NS5A/NS3-containing membranes, and does not colocalize with NS5A in HCV RNA-replicating cells. However, we find that the expression levels of Rab9 are 3-fold up-regulated in HCV replicon–expressing cells, suggesting that HCV infection may overall increase endosome-to-Golgi trafficking independently from TIP47. Future studies will further characterize TIP47's involvement in HCV infection, and the precise role of LD membranes in HCV RNA replication.

## Materials and Methods

### Plasmids

The N-terminal 31 amino acid–coding sequence of wild-type (genotype 1b, Con1) or W9A-mutant NS5A was cloned into the bait plasmid pBTM116 [44]. The TIP47 coding sequence was cloned into the prey plasmid pACT2 (Addgene plasmid 11343). The NS5A (genotype 1b, Con1) followed by a C-terminal Flag tag was cloned into the pEBB expression plasmid resulting in pNS5A-Flag. All mutant pNS5A-Flag plasmids were constructed by site-directed mutagenesis of pNS5A-Flag using the QuickChange II Site-Directed Mutagenesis Kit (Agilent Technologies). The expression plasmid for Myc-TIP47 was previously described [45]. Luciferase-Jc1 (genotype 2a), Luciferase-JFH1 (genotype 2a), Jc1 [46], and HCV-Replicon (genotype 1b, Con1) [47] have been described. pGL3.5 (expression vector for firefly luciferase) was purchased from Promega. All NS5A mutant HCV-Replicon plasmids were constructed by site-directed mutagenesis of HCV-Replicons using the QuickChange II XL Site-Directed Mutagenesis Kit (Agilent Technologies). ΔE-Jc1-NS5A-HA was constructed by deleting a portion of the E1/E2 genes from amino acids 313–567 in the Jc1 polyprotein and by inserting a XbaI/PmeI linker, followed by 3xHA and 6xHis tags into domain 3 of NS5A (at amino acid position 381, as described [48]). NS3–5B and NS3–5B W9A expression plasmids were constructed by cloning the NS3–5B region of the HCV-Replicon (Con1, genotype 1b) containing either wild-type or W9A mutant NS5A into the pEBB expression plasmid. Small hairpin RNAs targeting TIP47 (1445: GGGGCTCATTTCAAACTTA) or non-targeting small hairpin RNAs (GCCAAGAACGGTATCTGAA) were cloned into pSicoR (Addgene plasmid 11579) as described [49]. pMD2.G (Addgene plasmid 12259) pMDLg/pRRE (Addgene plasmid 12251) and pRSV-Rev (Addgene plasmid 12253) have been described [50].

### Cell lines and culture conditions

Huh7 and HEK293T cells were obtained from the American Type Culture Collection, Huh7.5 cells from Charles M. Rice, and Huh7 Lunet cells from Ralf Bartenschlager. All cells were grown under standard cell culture conditions and transfected with FuGENE6 (Roche) or X-tremeGENE9 (Roche), according to the manufacturer's protocol. Calcium phosphate-mediated transfection of HEK293T cells was used for the production of lentiviral particles. Huh7 Lunet cells were used for the microscopy studies due to their superior properties for immunofluorescence microscopy [51], if not noted otherwise. Viral RNA replication and infectious virus release properties are similar in both Huh7.5 and Huh7 Lunet cell lines [52].

### Antibodies and reagents

The following antibodies were obtained commercially and used as indicated (WB: Western Blot, IP: Immuno-precipitation, IF: Immunofluorescence microscopy): α-NS3 (abcam, #ab65407-100, WB), α-NS5A (Austral, #HCM-131-5, WB, IF), αNS5B (Abcam, #ab100895, WB), α-Calreticulin (Stressgen, SPA-600, WB), α-HA (Roche, 3F10, IF), α-Flag M2 (Sigma, #F-3165, IP, IF), α-Flag-HRP (Sigma, #A8592-2MG, WB), α-Flag (Sigma, # F7425, IP replicon cells) α-TIP47 (Abcam, ab47639, WB, IP), α-TIP47 (Sigma, #HPA006427, IF), α-myc-HRP (Santa Cruz, #sc-70463HRP, WB), α-HA (Roche, #3F10, IF), α-GAPDH (Abcam, #ab8245-100, WB), α-ADRP (Abcam, #ab37516, WB), α-Rab9 (Thermo Scientific, #MA3-067, WB, IF), α-Rab5 (Abcam, #ab18211, WB), α-Rab7 (Abcam, #ab50533, WB), α-mouse Alexa 488 (Invitrogen), α-rabbit Alexa 594 (Invitrogen), α-mouse Alexa 647 (Invitrogen), α-rat Alexa 647 (Invitrogen). Enzymes for molecular cloning were purchased from New England Biolabs; cell culture reagents, and Image IT-Fx-Signal Enhancer from Invitrogen; ProteinG-agarose and ProteinA-agarose from Sigma; and fine chemicals, if not noted otherwise, from Sigma.

### Yeast-Two-Hybrid Screen and Quantitative Yeast-Two-Hybrid Assay

The Yeast-2-Hybrid Screen was done using the MATCHMAKER Kit (Clontech) according to the manufacturer's recommendations.

A quantitative β-galactosidase assay was performed as described in the manual provided with the Clontech MATCHMAKER kit, which we used as an indirect assessment of the strength of interaction [53]. Briefly, yeasts were transformed with either bait plasmid, prey plasmid, or both, and plated out onto the appropriate media. Individual clones were inoculated into 5 ml of appropriate liquid media and cultured overnight at 30°C with shaking, after which 2 ml of this overnight culture was used to inoculate 6 ml of YPDA liquid media (1% yeast extract, 2% bactopeptone, 2% dextrose, 2% bacto agar), and further shaken at 30°C for 4 h. Cell extracts were then prepared and assayed for β-galactosidase activity. Units are expressed in terms of ΔA_420_ per minute per equivalent number of yeast cells.

### Immunoprecipitation and western blotting

For the immunoprecipitation experiments, Huh7 cells were transfected with plasmid DNA using Fugene6 (Roche) or X-tremeGene9 (Roche) according to the manufacturer's protocol. For immunoprecipitation experiments, transfected cells were lysed 48 h post-transfection in lysis buffer [150 mM NaCl, 1% NP-40, 1 mM EDTA, 50 mM Tris HCl, pH7.4 and protease inhibitor cocktail (Sigma)] for 30 min and passed 10 times through a G23 needle. Clarified lysates were pre-cleared with protein G agarose (Invitrogen) for 1 h at 4°C and after centrifugation, lysates were immunoprecipitated with protein G agarose (Invitrogen) and Flag antibody for 16 h at 4°C, washed five times in lysis buffer and resuspended in Laemmli buffer for SDS-PAGE. For the immunoprecipitation experiments with the replicon cell line, replicon cells were harvested and then processed as described above with minor changes. Protein A agarose, and anti-TIP47 antibodies for pull-down and anti-Flag antibodies as control were used. For chemiluminescent detection, we used SuperSignal West Pico or SuperSignal West Femto (Thermo Scientific) and ECL Hyperfilm (Amersham).

### Lentivirus production, transduction and western blotting

Lentiviral particles were produced as described [54]. Briefly, 293T cells were transfected with the pSicoR constructs, the two HIV-based packaging constructs pMDLg/pRRE and pRSV-Rev, and a construct expressing the glycoprotein of vesicular stomatitis virus pMD2.G. The culture supernatant containing pseudotyped lentiviral particles was concentrated by ultracentrifugation for 16 h at 20,000 rpm in a SW28 rotor. Infectious titers were determined by transducing cells with serial dilutions of the viral stocks and FACS analysis 2 days post-transduction. Transductions were carried out overnight at 37°C.

For western blot analysis of lentivirus-transduced cells, cells were lysed with RIPA buffer (1% NP-40, 10 mM Tris pH7.5, 150 mM NaCl, 1 mM EDTA) for 30 min at 4°C, and mixed 1∶1 with Laemmli buffer followed by SDS-PAGE.

### *In vitro* transcription of HCV RNA and transfection

Plasmids encoding HCV reporter viruses were linearized with PvuI (Jc1 and JFH1 viruses) or ScaI (HCV replicon), and purified by phenol-chloroform extraction. *In vitro* transcription and RNA purification was carried out using the MegaScript T7 kit (Ambion), according to the manufacturer's protocol. For RNA transfection, Huh7.5 or Huh7 Lunet cells were trypsinized, washed once in PBS, and resuspended in Dulbecco's Modified Eagle's Medium (DMEM) medium without serum at a concentration of 5×10^6^ cells/ml. 500 µl of cell suspension was mixed with 100 µl of DMEM, 10 µg HCV RNA and 5 µg of Myc-TIP47 plasmid DNA (where indicated) and pulsed at 220 V and 975 µF with the Gene Pulser II (Biorad).

### Luciferase Assay

After transfection with HCV RNA, Huh7.5 cells were seeded in 12-well plates. At indicated time points, the cells were lysed in 1x Passive Lysis Buffer (Promega). Luciferase activity was measured using Luciferase Assay Systems (Promega) on a MonoLight 2010 Luminometer (Pegasus Scientific Inc.).

### HCV-replicon assay and HCV-replicon cell line

1 µg of HCV replicon RNA and 1 µg of pGL3.5 (Firefly luciferase control plasmid) was transfected into Huh7.5 cells as described above. After transfection, cells were plated in 10 cm dishes for the replicon assay and into 12-well plates for the luciferase assay, and incubated at 37°C. To check for differences in transfection efficiencies, cells for the luciferase assay were lysed and luciferase activity was measured as described above. For the replicon assay, 24 h post-transfection 1 mg/ml G418 was added to the medium. Cells were kept under selective pressure for 3–4 weeks with media changed twice a week. Cells were then washed with PBS and stained with Crystal Violet. To establish an HCV replicon cell line, cells were kept under continuous G418 (800 µg/ml) pressure, and split twice a week.

### Immunofluorescence and LD staining

Cells grown on coverslips were fixed with 4% paraformaldehyde for 10 min at room temperature, washed with phosphate-buffered saline (PBS), and permeabilized either in 0.1% saponin in PBS (for TIP47) or 0.2% Triton X-100 in PBS (for Rab9) for 10 min. After incubation with Image IT-Fx-Signal Enhancer (Invitrogen) for 30 min, cells were washed with PBS and incubated in blocking solution (DMEM with 5% FBS +50% goat serum) for 20 min. Cells were washed and then incubated with primary antibodies either in PBS with 1% BSA (α-TIP47) or 0.2% Triton X-100/gelatin (α-Rab9) for 1 h, washed, and then incubated with secondary antibodies for 30 min. For oil-red-O (ORO) staining coverslips were incubated for 5 min in 60% isopropanol and stained with ORO staining solution (stock :0.5 g ORO (Sigma) in 100 ml of isopropanol; the stock was diluted 6∶4 (stock∶water) and filtered before use) and differentiated in 60% isopropanol for 1 sec. For BODIPY staining, fixed cells were stained for 10 min with 1 µg ml^–1^ BODIPY 493/503 (Molecular Probes) in PBS and then washed 3 times in PBS. For LipidToxRed Stain, fixed cells were stained for 30 min with LipidToxRed Neutral Stain (Molecular Probes, 1∶200) in PBS, and then washed 3 times in PBS. Coverslips were embedded in Mowiol (Calbiochem) mounting medium or in VECTASHIELD mounting medium with DAPI (Vector Laboratories).

### Epifluorescence microscopy and quantification of images

Cells were analyzed with Axio observer Z1 microscopy (Zeiss) equipped with EC Plan Neofluar 20x/0.5 PHM27, EC Plan Neofluar 40X/0.75 PH, and Plan Apo 63X/1.4 Oil DIC M27 objectives, filter sets 38HE, 43HE, 45, and 50, Optovar 1.25 and 1.6X magnification, and an Axiocam MRM REV 3. For quantification of LDs in cells or colocalization of proteins with LDs we used the automatic measurement program of the Volocity software (Perkin Elmer).

### Membrane flotation assay

Huh7.5 cells or HCV replicon cells (as described above) were trypsinized, washed and counted. 3×10^7^ cells total for each sample were resuspended in 3.5 ml PBS containing 0.25 M sucrose (PBS/sucrose) plus protease inhibitor cocktail (Sigma). The cells were then lysed with 200 passages in a tight fitting dounce homogenizer (Wheaton) to ensure approximately 90% lysis. The cell lysate was then spun at 2500× g for 10 min at 4°C to pellet cellular debris and nuclei. The resulting supernatant was referred to as crude lysate. Equal amounts of protein (5 mg) for each cell type was adjusted in volume to 2 ml with PBS/sucrose and mixed with 2 ml of 60% iodixanol (Sigma) resulting in a 30% iodixanol concentration. A discontinuous iodixanol gradient (10%, 20%) was layered on top of the lysate/iodixanol mixture, and the gradient was spun at 200,000× g for 16 h at 4°C in a SW41T Rotor. A total of 22 fractions (500 µl each) were collected from top to bottom. The fractions were mixed 1∶1 with Laemmli buffer for SDS-PAGE. Quantification of protein expression in western blots was done in ImageJ Software.

### RNA extraction and quantification by real-time RT-PCR

20 ug of carrier RNA (Qiagen) was added to 150 ul of each fraction. RNA was extracted from each fraction using the Quick-RNA MiniPrep kit (Zymo) according to manufacturer's recommendations. RNA was eluted using 35 ul H_2_O.

cDNA was synthesized using an HCV replicon (Con1, genotype 1b) antisense probe (nt 283-302; 5′-ctttcgcgacccaacactac-3′) and AMV reverse transcriptase (Promega) followed by RNase A (Thermo Scientific) digestion. For the generation of RNA standards, cDNA was generated for a 2-fold serial dilution of in vitro transcribed HCV replicon RNAs using the above protocol.

For real-time quantitative PCR, we used HCV replicon specific primers (sense (13–30): 5′-acgactcactatagccag-3′, anti-sense (268–285): 5′-tactcggctagcagtctc-3′). Real-time PCR was performed using a 2x HotSybr Real-time PCR kit (MCLAB) on a 7900HT Fast Real-time RT-PCR System (Applied Biosystems). Each sample was done in triplicates. The cDNA from the 2-fold serial dilution of HCV Replicon RNA was used to create a standard curve. Absolute amounts of viral RNA in fractions were calculated using this standard curve.

### Lipid droplet isolation

Lipid droplets were isolated as described with minor changes [12]. Briefly, cells were scraped in PBS, lysed in hypotonic buffer (50 mM HEPES, 1 mM EDTA and 2 mM MgCl2, pH 7.4) supplemented with protease inhibitors with 30 strokes in a tight-fitting Dounce homogenizer. After spinning 5 min at 1500 rpm, post-nuclear fractions were mixed with equal volumes of 1.5 M sucrose in isotonic buffer (50 mM HEPES, 100 mM KCl, 2 mM MgCl2) and placed at the bottom of SW55 Ti (Beckman) centrifuge tubes, overlaid with isotonic buffer containing 1 mM PMSF and centrifuged for 2 h at 100,000 g. Tubes were frozen at -80°C for 1 h. The top layer of floating lipid droplet fraction was cut off, and proteins of this fraction were precipitated with 15% trichloroacetic acid and 30% acetone, washed once with acetone and resuspended in urea loading dye (200 mM Tris/HCl, pH 6.8, 8 M urea, 5% SDS, 1 mM EDTA, 0.1% bromophenol blue, 15 mM DTT).

#### CellTiter-Blue Cell Viability assay

The CellTiter-Blue Cell Viability Assay (Promega) was performed according to the manufacturer's recommendations. Briefly, naïve Huh7.5, and Huh7.5 containing shRNA Control or shRNA TIP47 were seeded in a 2-fold serial dilution in 96-well plates in 100 ul medium. Cells were incubated at 37°C for 1 hour. 20 ul CellTiter-Blue Reagent was added to each well, and the plates then further incubated at 37°C for 18 h. Absorbance was measured at 570 nm and 600 nm. Absorbance at 600 nm of medium only was deducted from absorbance at 570 nm of each time point.

### Statistical analysis

Statistical analysis was performed using unpaired two-tailed student's t-test.

### Accession numbers

The NCBI gene database (http://www.ncbi.nlm.nih.gov/gene) ID numbers for genes and transcripts in this paper are TIP47 (ID# 10226), Perilipin (ID# 5346), ADRP (ID# 123), MPR (ID# 3482), Rab9 (ID# 9367), Calreticulin (ID# 811), Rab5 (ID# 5868), Rab7 (ID#7879).

## Supporting Information

Figure S1 **Localization of NS5A-Flag and W9A-NS5A-Flag in Huh7 Lunet cells.** Indirect immunofluorescence of NS5A (green) in Huh7 Lunet cells transfected with DNA expression vectors for either NS5A-Flag, W9A-NS5A-Flag, or Δ31-NS5A-Flag (containing an N-terminal 31 amino acids deletion of NS5A). Lipid droplets were stained with LipidToxRed (red).
(TIF)

Figure S2 **Effect of shRNA on cell viability and LD morphology.** A) CellTiter Blue Cell Viability assay: Naïve Huh7.5, or Huh7.5 containing shRNA Control or shRNA TIP47 were seeded in a 2-fold serial dilution in 96-well plates Cells were incubated at 37°C for 1 hour. CellTiter-Blue Reagent was added to each well, and the plates then further incubated at 37°C for 18 h. Absorbance was measured at 570 nm and 600 nm. Absorbance at 600 nm of medium only was deducted from absorbance at 570 nm of each time point. B) Number of LDs per cell in Huh7.5 cells transduced with either Ctr shRNA or shRNA targeting TIP47. Number of LDs were quantified using the automatic measurement program of Volocity software by quantifying RedO stain of LDs in cells. C) Mean diameter of LDs per cell. Diameter of LDs were quantified using the automatic measurement program of Volocity software by quantifying RedO stain of LDs in cells.
(TIF)

Figure S3 **Expression and distribution of cellular Rab proteins during HCV RNA replication.** A) Western Blot of membrane flotation assay as described above (Figure 4A). Fractions collected were assessed by western blot with antibodies specific for Rab9, Rab7, or Rab5. CL = total cell lysate before gradient centrifugation. B) Profile of protein expression from western blot in (A). Protein expression is shown as protein amount (as measured by intensity) in one fraction as a percentage of total protein amount in all 22 fractions combined. Protein quantification was done in ImageJ. C) Indirect immunofluorescence of Rab9 (green) and NS5A (HA, red) in either non-transfected Huh7 Lunet cells or cells transfected with ΔE-Jc1-NS5A-HA RNA (a monocistronic Jc1 RNA containing a 3x HA-tagged NS5A within the open reading frame, and a deletion of E1/E2 proteins). Lipid droplets were stained with LipidToxRed (shown in white). The choice of viral construct was determined by the species of the antibodies available in order to do a triple stain of Rab9, NS5A and LDs.
(TIF)

## References

[1] Egger D, Wolk B, Gosert R, Bianchi L, Blum HE, et al (2002) Expression of hepatitis C virus proteins induces distinct membrane alterations including a candidate viral replication complex. J Virol 76: 5974–5984. 12021330 10.1128/JVI.76.12.5974-5984.2002 PMC136238

[2] Bartenschlager R, Frese M, Pietschmann T (2004) Novel insights into hepatitis C virus replication and persistence. Adv Virus Res 63: 71–180. 15530561 10.1016/S0065-3527(04)63002-8

[3] Gosert R, Egger D, Lohmann V, Bartenschlager R, Blum HE, et al (2003) Identification of the hepatitis C virus RNA replication complex in Huh-7 cells harboring subgenomic replicons. J Virol 77: 5487–5492. 12692249 10.1128/JVI.77.9.5487-5492.2003 PMC153965

[4] Macdonald A, Harris M (2004) Hepatitis C virus NS5A: tales of a promiscuous protein. J Gen Virol 85: 2485–2502. 15302943 10.1099/vir.0.80204-0

[5] Buhler S, Bartenschlager R (2012) New targets for antiviral therapy of chronic hepatitis C. Liver Int 32 Suppl 1: 9–16. 22212566 10.1111/j.1478-3231.2011.02701.x

[6] Alvisi G, Madan V, Bartenschlager R (2011) Hepatitis C virus and host cell lipids: an intimate connection. RNA Biol 8: 258–269. 21593584 10.4161/rna.8.2.15011

[7] He Y, Staschke KA, Tan SL (2006) HCV NS5A: A Multifunctional Regulator of Cellular Pathways and Virus Replication. In: Tan SL, editor. Hepatitis C Viruses: Genomes and Molecular Biology. Norfolk, UK. 21250384

[8] Moriishi K, Matsuura Y (2012) Exploitation of lipid components by viral and host proteins for hepatitis C virus infection. Front Microbiol 3: 54. 22347882 10.3389/fmicb.2012.00054 PMC3278987

[9] Brass V, Bieck E, Montserret R, Wolk B, Hellings JA, et al (2002) An amino-terminal amphipathic alpha-helix mediates membrane association of the hepatitis C virus nonstructural protein 5A. J Biol Chem 277: 8130–8139. 11744739 10.1074/jbc.M111289200

[10] Elazar M, Cheong KH, Liu P, Greenberg HB, Rice CM, et al (2003) Amphipathic helix-dependent localization of NS5A mediates hepatitis C virus RNA replication. J Virol 77: 6055–6061. 12719597 10.1128/JVI.77.10.6055-6061.2003 PMC154017

[11] Penin F, Brass V, Appel N, Ramboarina S, Montserret R, et al (2004) Structure and function of the membrane anchor domain of hepatitis C virus nonstructural protein 5A. J Biol Chem 279: 40835–40843. 15247283 10.1074/jbc.M404761200

[12] Miyanari Y, Atsuzawa K, Usuda N, Watashi K, Hishiki T, et al (2007) The lipid droplet is an important organelle for hepatitis C virus production. Nat Cell Biol 9: 1089–1097. 17721513 10.1038/ncb1631

[13] Evans MJ, Rice CM, Goff SP (2004) Phosphorylation of hepatitis C virus nonstructural protein 5A modulates its protein interactions and viral RNA replication. Proc Natl Acad Sci U S A 101: 13038–13043. 15326295 10.1073/pnas.0405152101 PMC516513

[14] Appel N, Zayas M, Miller S, Krijnse-Locker J, Schaller T, et al (2008) Essential role of domain III of nonstructural protein 5A for hepatitis C virus infectious particle assembly. PLoS Pathog 4: e1000035. 18369481 10.1371/journal.ppat.1000035 PMC2268006

[15] Tellinghuisen TL, Foss KL, Treadaway J (2008) Regulation of hepatitis C virion production via phosphorylation of the NS5A protein. PLoS Pathog 4: e1000032. 18369478 10.1371/journal.ppat.1000032 PMC2265800

[16] Murphy DJ (2001) The biogenesis and functions of lipid bodies in animals, plants and microorganisms. Prog Lipid Res 40: 325–438. 11470496 10.1016/s0163-7827(01)00013-3

[17] Bickel PE, Tansey JT, Welte MA (2009) PAT proteins, an ancient family of lipid droplet proteins that regulate cellular lipid stores. Biochim Biophys Acta 1791: 419–440. 19375517 10.1016/j.bbalip.2009.04.002 PMC2782626

[18] Wolins NE, Rubin B, Brasaemle DL (2001) TIP47 associates with lipid droplets. J Biol Chem 276: 5101–5108. 11084026 10.1074/jbc.M006775200

[19] Gross DN, Miyoshi H, Hosaka T, Zhang HH, Pino EC, et al (2006) Dynamics of lipid droplet-associated proteins during hormonally stimulated lipolysis in engineered adipocytes: stabilization and lipid droplet binding of adipocyte differentiation-related protein/adipophilin. Mol Endocrinol 20: 459–466. 16239256 10.1210/me.2005-0323

[20] Xu G, Sztalryd C, Lu X, Tansey JT, Gan J, et al (2005) Post-translational regulation of adipose differentiation-related protein by the ubiquitin/proteasome pathway. J Biol Chem 280: 42841–42847. 16115879 10.1074/jbc.M506569200

[21] Aivazian D, Serrano RL, Pfeffer S (2006) TIP47 is a key effector for Rab9 localization. J Cell Biol 173: 917–926. 16769818 10.1083/jcb.200510010 PMC2063917

[22] Diaz E, Pfeffer SR (1998) TIP47: a cargo selection device for mannose 6-phosphate receptor trafficking. Cell 93: 433–443. 9590177 10.1016/s0092-8674(00)81171-x

[23] Riederer MA, Soldati T, Shapiro AD, Lin J, Pfeffer SR (1994) Lysosome biogenesis requires Rab9 function and receptor recycling from endosomes to the trans-Golgi network. J Cell Biol 125: 573–582. 7909812 10.1083/jcb.125.3.573 PMC2119986

[24] Bulankina AV, Deggerich A, Wenzel D, Mutenda K, Wittmann JG, et al (2009) TIP47 functions in the biogenesis of lipid droplets. J Cell Biol 185: 641–655. 19451273 10.1083/jcb.200812042 PMC2711566

[25] Lohmann V, Korner F, Koch J, Herian U, Theilmann L, et al (1999) Replication of subgenomic hepatitis C virus RNAs in a hepatoma cell line. Science 285: 110–113. 10390360 10.1126/science.285.5424.110

[26] Berger KL, Cooper JD, Heaton NS, Yoon R, Oakland TE, et al (2009) Roles for endocytic trafficking and phosphatidylinositol 4-kinase III alpha in hepatitis C virus replication. Proc Natl Acad Sci U S A 106: 7577–7582. 19376974 10.1073/pnas.0902693106 PMC2678598

[27] Krieger N, Lohmann V, Bartenschlager R (2001) Enhancement of hepatitis C virus RNA replication by cell culture-adaptive mutations. J Virol 75: 4614–4624. 11312331 10.1128/JVI.75.10.4614-4624.2001 PMC114214

[28] Kuiken C, Yusim K, Boykin L, Richardson R (2005) The Los Alamos hepatitis C sequence database. Bioinformatics 21: 379–384. 15377502 10.1093/bioinformatics/bth485

[29] Yeaman C, Grindstaff KK, Wright JR, Nelson WJ (2001) Sec6/8 complexes on trans-Golgi network and plasma membrane regulate late stages of exocytosis in mammalian cells. J Cell Biol 155: 593–604. 11696560 10.1083/jcb.200107088 PMC2198873

[30] Wolins NE, Brasaemle DL, Bickel PE (2006) A proposed model of fat packaging by exchangeable lipid droplet proteins. FEBS Lett 580: 5484–5491. 16962104 10.1016/j.febslet.2006.08.040

[31] Kolesnikova L, Bamberg S, Berghofer B, Becker S (2004) The matrix protein of Marburg virus is transported to the plasma membrane along cellular membranes: exploiting the retrograde late endosomal pathway. J Virol 78: 2382–2393. 14963134 10.1128/JVI.78.5.2382-2393.2004 PMC369247

[32] Liu P, Bartz R, Zehmer JK, Ying YS, Zhu M, et al (2007) Rab-regulated interaction of early endosomes with lipid droplets. Biochim Biophys Acta 1773: 784–793. 17395284 10.1016/j.bbamcr.2007.02.004 PMC2676670

[33] Manna D, Aligo J, Xu C, Park WS, Koc H, et al (2010) Endocytic Rab proteins are required for hepatitis C virus replication complex formation. Virology 398: 21–37. 20005553 10.1016/j.virol.2009.11.034 PMC2823978

[34] Negro F, Sanyal AJ (2009) Hepatitis C virus, steatosis and lipid abnormalities: clinical and pathogenic data. Liver Int 29 Suppl 2: 26–37. 19187070 10.1111/j.1478-3231.2008.01950.x

[35] Negro F (2004) Hepatitis C virus and liver steatosis: when fat is not beautiful. J Hepatol 40: 533–535. 15123371 10.1016/j.jhep.2004.01.011

[36] Roingeard P, Hourioux C, Blanchard E, Prensier G (2008) Hepatitis C virus budding at lipid droplet-associated ER membrane visualized by 3D electron microscopy. Histochem Cell Biol 130: 561–566. 18512067 10.1007/s00418-008-0447-2

[37] Heaton NS, Randall G (2011) Dengue virus and autophagy. Viruses 3: 1332–1341. 21994782 10.3390/v3081332 PMC3185800

[38] Saad JS, Miller J, Tai J, Kim A, Ghanam RH, et al (2006) Structural basis for targeting HIV-1 Gag proteins to the plasma membrane for virus assembly. Proc Natl Acad Sci U S A 103: 11364–11369. 16840558 10.1073/pnas.0602818103 PMC1544092

[39] Parent LJ, Bennett RP, Craven RC, Nelle TD, Krishna NK, et al (1995) Positionally independent and exchangeable late budding functions of the Rous sarcoma virus and human immunodeficiency virus Gag proteins. J Virol 69: 5455–5460. 7636991 10.1128/jvi.69.9.5455-5460.1995 PMC189393

[40] Lopez-Verges S, Camus G, Blot G, Beauvoir R, Benarous R, et al (2006) Tail-interacting protein TIP47 is a connector between Gag and Env and is required for Env incorporation into HIV-1 virions. Proc Natl Acad Sci U S A 103: 14947–14952. 17003132 10.1073/pnas.0602941103 PMC1595456

[41] Carvalho FA, Carneiro FA, Martins IC, Assuncao-Miranda I, Faustino AF, et al (2012) Dengue virus capsid protein binding to hepatic lipid droplets (LD) is potassium ion dependent and is mediated by LD surface proteins. J Virol 86: 2096–2108. 22130547 10.1128/JVI.06796-11 PMC3302401

[42] Samsa MM, Mondotte JA, Iglesias NG, Assuncao-Miranda I, Barbosa-Lima G, et al (2009) Dengue virus capsid protein usurps lipid droplets for viral particle formation. PLoS Pathog 5: e1000632. 19851456 10.1371/journal.ppat.1000632 PMC2760139

[43] Chen Y, Honeychurch KM, Yang G, Byrd CM, Harver C, et al (2009) Vaccinia virus p37 interacts with host proteins associated with LE-derived transport vesicle biogenesis. Virol J 6: 44. 19400954 10.1186/1743-422X-6-44 PMC2685784

[44] Bartel P, Chien CT, Sternglanz R, Fields S (1993) Elimination of false positives that arise in using the two-hybrid system. Biotechniques 14: 920–924. 8333960

[45] Sincock PM, Ganley IG, Krise JP, Diederichs S, Sivars U, et al (2003) Self-assembly is important for TIP47 function in mannose 6-phosphate receptor transport. Traffic 4: 18–25. 12535272 10.1034/j.1600-0854.2003.40104.x

[46] Pietschmann T, Kaul A, Koutsoudakis G, Shavinskaya A, Kallis S, et al (2006) Construction and characterization of infectious intragenotypic and intergenotypic hepatitis C virus chimeras. Proc Natl Acad Sci U S A 103: 7408–7413. 16651538 10.1073/pnas.0504877103 PMC1455439

[47] Choi J, Lee KJ, Zheng Y, Yamaga AK, Lai MM, et al (2004) Reactive oxygen species suppress hepatitis C virus RNA replication in human hepatoma cells. Hepatology 39: 81–89. 14752826 10.1002/hep.20001

[48] Schaller T, Appel N, Koutsoudakis G, Kallis S, Lohmann V, et al (2007) Analysis of hepatitis C virus superinfection exclusion by using novel fluorochrome gene-tagged viral genomes. J Virol 81: 4591–4603. 17301154 10.1128/JVI.02144-06 PMC1900174

[49] Ventura A, Meissner A, Dillon CP, McManus M, Sharp PA, et al (2004) Cre-lox-regulated conditional RNA interference from transgenes. Proc Natl Acad Sci U S A 101: 10380–10385. 15240889 10.1073/pnas.0403954101 PMC478580

[50] Dull T, Zufferey R, Kelly M, Mandel RJ, Nguyen M, et al (1998) A third-generation lentivirus vector with a conditional packaging system. J Virol 72: 8463–8471. 9765382 10.1128/jvi.72.11.8463-8471.1998 PMC110254

[51] Shavinskaya A, Boulant S, Penin F, McLauchlan J, Bartenschlager R (2007) The lipid droplet binding domain of hepatitis C virus core protein is a major determinant for efficient virus assembly. J Biol Chem 282: 37158–37169. 17942391 10.1074/jbc.M707329200

[52] Koutsoudakis G, Herrmann E, Kallis S, Bartenschlager R, Pietschmann T (2007) The level of CD81 cell surface expression is a key determinant for productive entry of hepatitis C virus into host cells. J Virol 81: 588–598. 17079281 10.1128/JVI.01534-06 PMC1797465

[53] Felinski EA, Quinn PG (1999) The CREB constitutive activation domain interacts with TATA-binding protein-associated factor 110 (TAF110) through specific hydrophobic residues in one of the three subdomains required for both activation and TAF110 binding. J Biol Chem 274: 11672–11678. 10206980 10.1074/jbc.274.17.11672

[54] Naldini L, Blomer U, Gallay P, Ory D, Mulligan R, et al (1996) In vivo gene delivery and stable transduction of nondividing cells by a lentiviral vector. Science 272: 263–267. 8602510 10.1126/science.272.5259.263

