# Supplementary figures and images for: Lipid Droplet-Binding Protein TIP47 Regulates Hepatitis C Virus RNA Replication through Interaction with the Viral NS5A Protein
Source: PLoS Pathog. 2013 Apr 11;9(4):e1003302. doi: 10.1371/journal.ppat.1003302 (PMC3623766; doi:10.1371/journal.ppat.1003302)

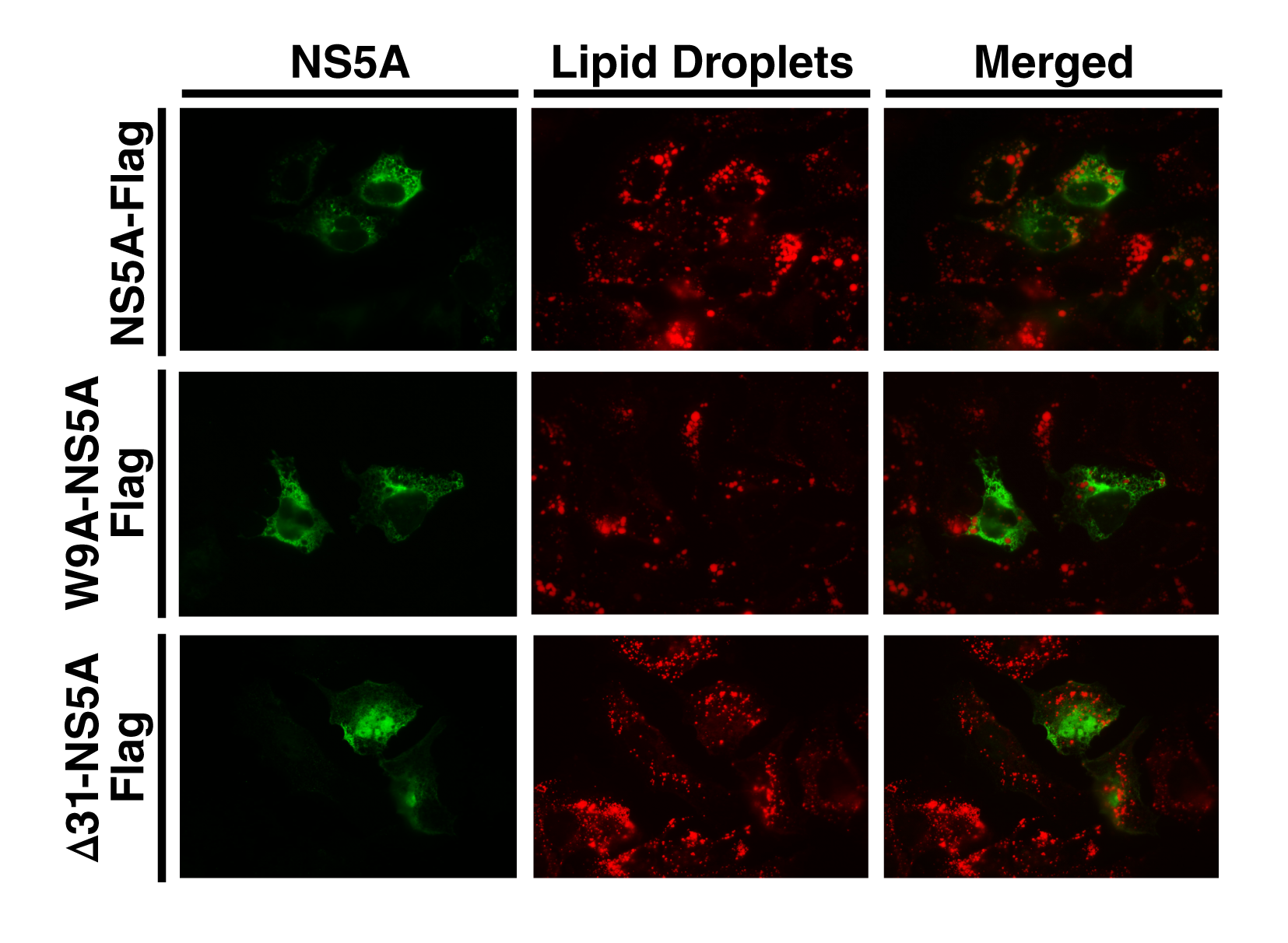

Supplement: Figure S1 — Localization of NS5A-Flag and W9A-NS5A-Flag in Huh7 Lunet cells. Indirect immunofluorescence of NS5A (green) in Huh7 Lunet cells transfected with DNA expression vectors for either NS5A-Flag, W9A-NS5A-Flag, or Δ31-NS5A-Flag (containing an N-terminal 31 amino acids deletion of NS5A). Lipid droplets were stained with LipidToxRed (red). (TIF) [file ppat.1003302.s001.tif]

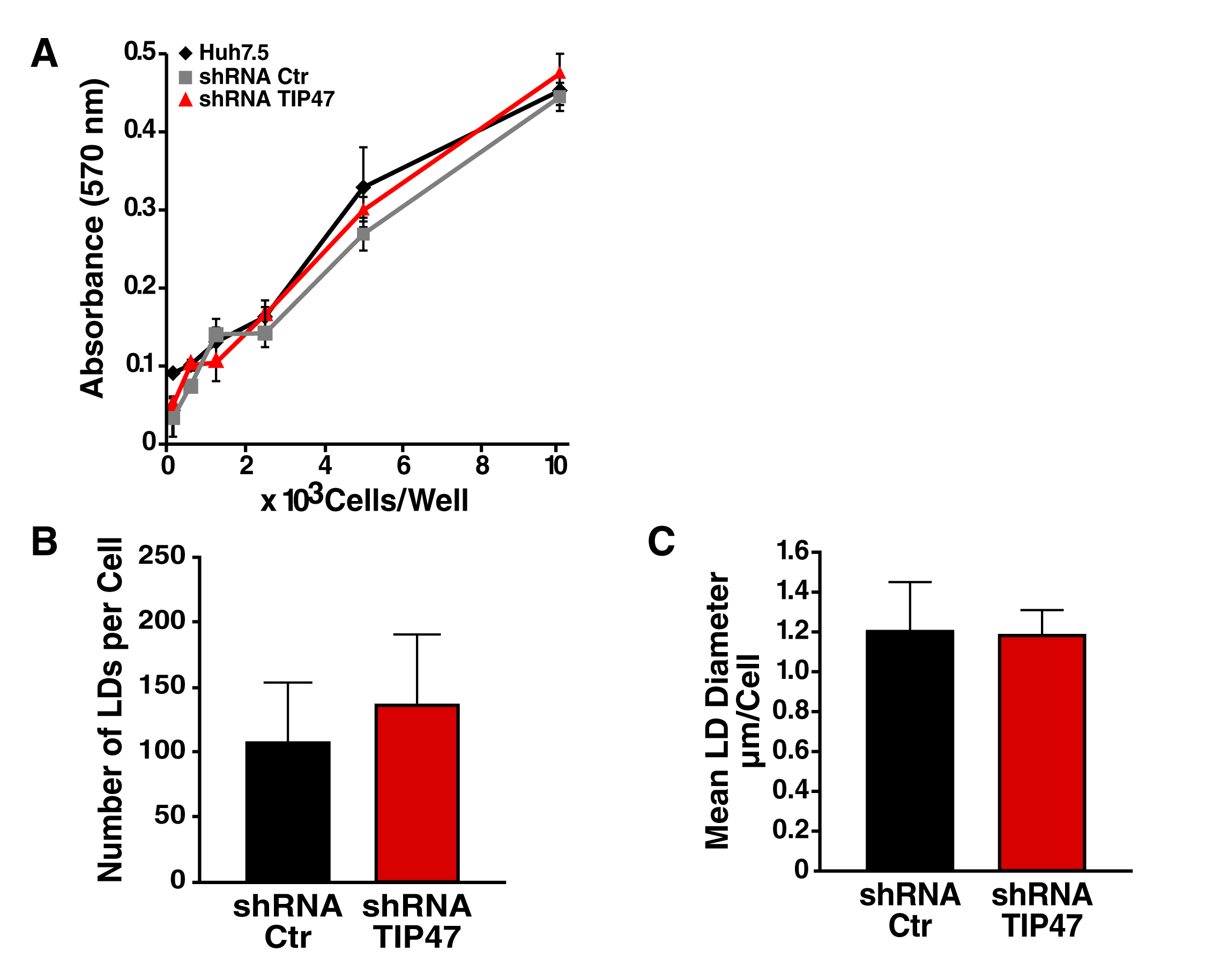

Supplement: Figure S2 — Effect of shRNA on cell viability and LD morphology. A) CellTiter Blue Cell Viability assay: Naïve Huh7.5, or Huh7.5 containing shRNA Control or shRNA TIP47 were seeded in a 2-fold serial dilution in 96-well plates Cells were incubated at 37°C for 1 hour. CellTiter-Blue Reagent was added to each well, and the plates then further incubated at 37°C for 18 h. Absorbance was measured at 570 nm and 600 nm. Absorbance at 600 nm of medium only was deducted from absorbance at 570 nm of each time point. B) Number of LDs per cell in Huh7.5 cells transduced with either Ctr shRNA or shRNA targeting TIP47. Number of LDs were quantified using the automatic measurement program of Volocity software by quantifying RedO stain of LDs in cells. C) Mean diameter of LDs per cell. Diameter of LDs were quantified using the automatic measurement program of Volocity software by quantifying RedO stain of LDs in cells. (TIF) [file ppat.1003302.s002.tif]

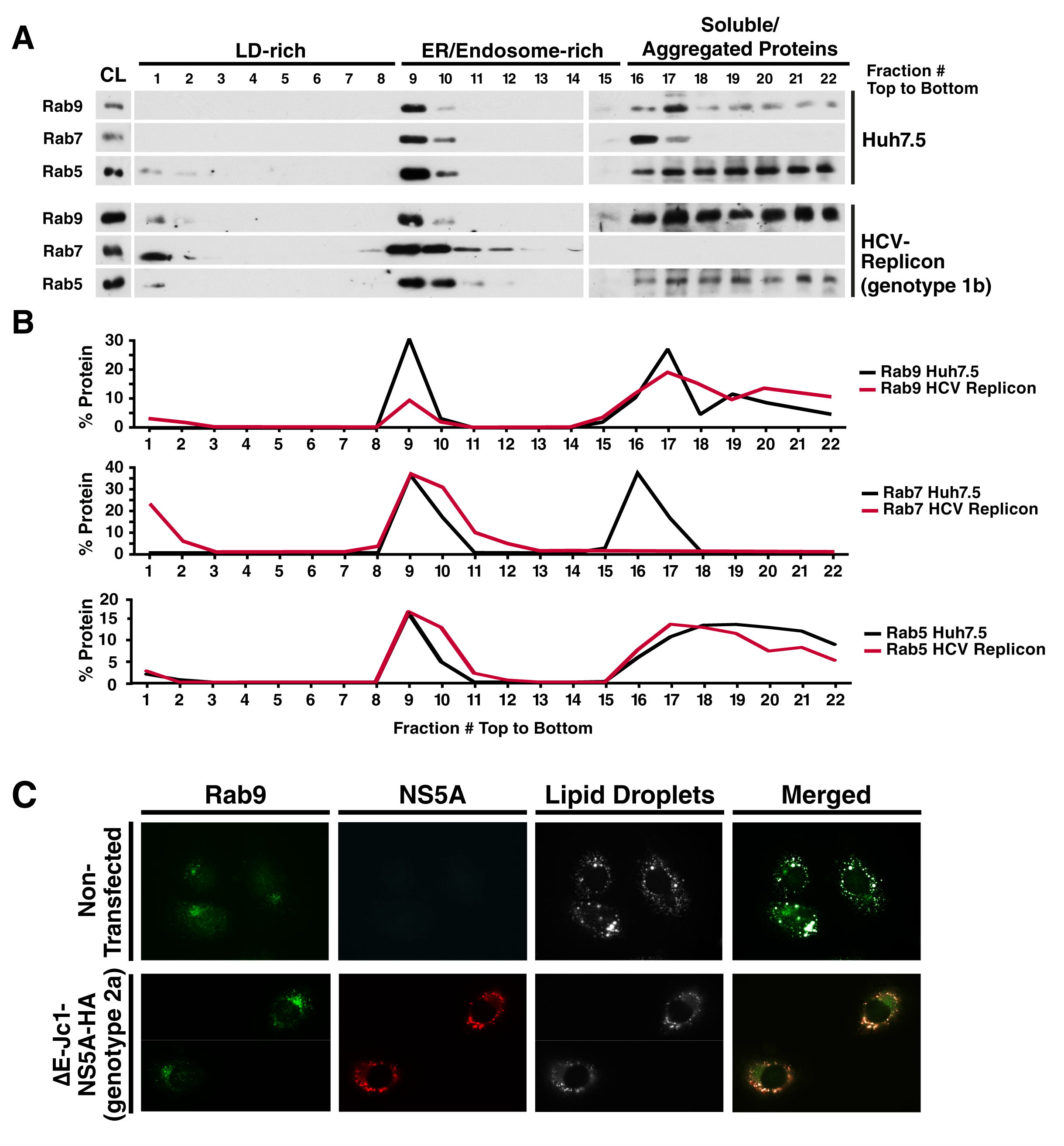

Supplement: Figure S3 — Expression and distribution of cellular Rab proteins during HCV RNA replication. A) Western Blot of membrane flotation assay as described above (Figure 4A). Fractions collected were assessed by western blot with antibodies specific for Rab9, Rab7, or Rab5. CL = total cell lysate before gradient centrifugation. B) Profile of protein expression from western blot in (A). Protein expression is shown as protein amount (as measured by intensity) in one fraction as a percentage of total protein amount in all 22 fractions combined. Protein quantification was done in ImageJ. C) Indirect immunofluorescence of Rab9 (green) and NS5A (HA, red) in either non-transfected Huh7 Lunet cells or cells transfected with ΔE-Jc1-NS5A-HA RNA (a monocistronic Jc1 RNA containing a 3x HA-tagged NS5A within the open reading frame, and a deletion of E1/E2 proteins). Lipid droplets were stained with LipidToxRed (shown in white). The choice of viral construct was determined by the species of the antibodies available in order to do a triple stain of Rab9, NS5A and LDs. (TIF) [file ppat.1003302.s003.tif]
